# Rapid Differential Detection of Abrin Isoforms by an Acetonitrile- and Ultrasound-Assisted On-Bead Trypsin Digestion Coupled with LC-MS/MS Analysis

**DOI:** 10.3390/toxins13050358

**Published:** 2021-05-18

**Authors:** Long-Hui Liang, Yang Yang, Shu Geng, Xi Cheng, Hui-Lan Yu, Chang-Cai Liu, Shi-Lei Liu

**Affiliations:** 1 State Key Laboratory of NBC Protection for Civilian, Beijing 102205, China; llhkingdom@163.com (L.-H.L.); ricdyihui@163.com (Y.Y.); gshu12@163.com (S.G.); yuhuilan1@163.com (H.-L.Y.); 2 The Laboratory of Analytical Chemistry, Research Institute of Chemical Defence, Beijing 102205, China; gaysci_C4@outlook.com

**Keywords:** marker peptides, RIP II protein toxins, detection of abrin, HPLC-ESI-MS/MS, ultrasound-assisted trypsin digestion

## Abstract

The high toxic abrin from the plant *Abrus precatorius* is a type II ribosome-inactivating protein toxin with a human lethal dose of 0.1–1.0 µg/kg body weight. Due to its high toxicity and the potential misuse as a biothreat agent, it is of great importance to developing fast and reliable methods for the identification and quantification of abrin in complex matrices. Here, we report rapid and efficient acetonitrile (ACN)- and ultrasound-assisted on-bead trypsin digestion method combined with HPLC-MS/MS for the quantification of abrin isoforms in complex matrices. Specific peptides of abrin isoforms were generated by direct ACN-assisted trypsin digestion and analyzed by HPLC-HRMS. Combined with in silico digestion and BLASTp database search, fifteen marker peptides were selected for differential detection of abrin isoforms. The abrin in milk and plasma was enriched by immunomagnetic beads prepared by biotinylated anti-abrin polyclonal antibodies conjugated to streptavidin magnetic beads. The ultrasound-assisted on-bead trypsin digestion method was carried out under the condition of 10% ACN as denaturant solvent, the entire digestion time was further shortened from 90 min to 30 min. The four peptides of T3A^a,b,c,d^, T12A^a^, T15A^b,^ and T9A^c,d^ were chosen as quantification for total abrin, abrin-a, abrin-b, and abrin-c/d, respectively. The absolute quantification of abrin and its isoforms was accomplished by isotope dilution with labeled AQUA peptides and analyzed by HPLC-MS/MS (MRM). The developed method was fully validated in milk and plasma matrices with quantification limits in the range of 1.0-9.4 ng/mL for the isoforms of abrin. Furthermore, the developed approach was applied for the characterization of abrin isoforms from various fractions from gel filtration separation of the seeds, and measurement of abrin in the samples of biotoxin exercises organized by the Organization for the Prohibition of Chemical Weapons (OPCW). This study provided a recommended method for the differential identification of abrin isoforms, which are easily applied in international laboratories to improve the capabilities for the analysis of biotoxin samples.

## 1. Introduction

Abrin from *Abrus precatorius* (*A*. *precatorius*) together with ricin belongs to the type II ribosome-inactivating protein (RIP-II) which consists of an A-chain with N-glycosidase activity and a B-chain with lectin activity. The two chains are connected by a disulfide bond. The B chain can bind to the galactose receptor on the cell membrane, allowing the A-chain to enter the cell through endocytosis. The A chain irreversibly inactivates the large ribosomal subunit by deadenylating a specific adenine from 28S rRNA, and arrests the protein synthesis, thereby releasing cytotoxicity [1]. Abrin is one of the most toxic plant toxins, and its toxicity is about 70 times that of ricin as a potentially lethal biotoxin agent in the Chemical Weapons Convention [2,3]. 

The content of the natural abrin of the seeds of *A. precatorius* is 0.1 to 0.8% [4,5], which less than that of ricin in the seeds of *Ricinus communis* (1–5 g/100 g of oil-deprived seed mush) [6]. The intravenous injection lethal doses of abrin are 0.04 µg/kg and 0.1–1.0 µg/kg for mice and humans [4,7]. Based on its extreme toxicity, wide availability, and ease of preparation, abrin has similar potential hazards with ricin and the measurement of abrin is also being taken seriously. Different from two isoforms of ricin, four isoforms of abrin (abrin-a, abrin-b, abrin-c, and abrin-d) have been isolated from the seeds of *A. precatorius*, with a relative molecular mass range between 63–67 kDa [4]. Although about 78% amino acid sequence similarity is found among the four isoforms of abrin, they exhibit significantly different biochemical properties such as the ability to bind galactose and cytotoxicity [4,5]. 

The lethal doses of four abrin isoforms range from 10, 25, 16, and 31 μg/kg (intraperitoneal injection) [4]. In addition, abrin shares high amino acid sequence similarity with Abrus agglutinin (67% in A-chain and 80% in B-chain) which is also present in the castor bean. However, the toxicity of Abrus agglutinin is several magnitudes less than abrin due to structural changes in the active site [5,8]. In addition, the relative content of different abrin subtypes in different abrin species is different. Therefore, differential identification of different isoforms of abrin can provide traceability information of abrin species origin or variety.

Over the last few decades, numerous methods for the detection of ricin have been developed. However, only a few techniques were reported for the detection of abrin, most of which are mainly based on antibody-based immunoassay technologies such as enzyme-linked immunosorbent assay [9] and western blot [10]. Although immunoassays are sensitive and quantitative, they easily generate false-positive results due to the nonspecific binding of abrin antibodies against its homologous protein of *Abrus* agglutinin. So far, no antibodies of abrin have been found to specially recognize one of four abrin isomers, resulting in no differential detection of abrin isoforms using the immunoassay-based method. In addition, some functional-based assays were developed by measurement of the N-glycosidase activity of abrin [11,12]. However, it is difficult to achieve the differential identification of abrin and other RIP-II toxins because they share the common depurination activity. Notably, the unambiguous identification and quantification of bioterrorist-related toxins in complex matrices can be achieved using the method of proteinase digestion combined with mass spectrometry analysis, which were based on the amino acid sequence difference of the resulting specific peptides [13,14,15,16,17]. However, as for the unambiguous identification of abrin isoforms, it remains challenging and relies on the proper selection of marker peptides from proteinase digestion. Some problems of them had been addressed by a developed quantification method of abrin and its isoforms in complex matrices [18], in which immunomagnetic beads enrichment and an ultrasound-assisted digestion procedure were used to shorten the digestion time, and a panel of common and isoform-specific peptides were selected for the identification of abrin and its isoforms [18]. Despite this, this method could only make quantification of total abrin, abrin-a, and abrin-b, c, d as the marker peptides from abrin-b and abrin-c, d were not specially selected as the quantification peptides. The method needed one additional procedure for degrading RapiGest SF surfactant prior to LC-MS/MS analysis, in which RapiGest SF was used to promote the protein unfolding before digestion. 

To address these, herein an improved approach for the quantification of abrin and its isoforms was developed using HPLC-MS/MS detection of the marker peptides from tryptic digestion for total abrin, abrin-a, abrin-b, and abrin-c,d, respectively. Fifteen common or isoform-specific peptides with high and stable yields are obtained as marker peptides for unambiguous identification of abrin and its isoforms using our direct ACN-assisted trypsin digestion method developed previously [17]. Combined with immunomagnetic bead enrichment, marker peptides of total abrin, abrin-a, abrin-b, and abrin-c/d are quantified in a single experiment. The ultrasound-assisted on-bead trypsin digestion method was improved by the addition of 10% of ACN as protein denaturant instead of RapiGest SF, which further reduced the total digestion time from 90 min to 30 min. This developed method allows for the rapid identification and quantification of abrin and its isoforms in complex matrices. It has been successfully applied for the unambiguous identification of abrin samples from the OPCW biotoxin exercises.

## 2. Results and Discussion

### 2.1. Screening and Identification of Specific Marker Peptides for Each Abrin Isoform 

The uniqueness of tryptic peptides of four abrin isoforms was identified by BLAST search across NCBI non-redundant database following in silico tryptic digestion. As a result, there were nine specific peptides of T1A^a^, T2A^a^, T6A^a^, T9A^a^, T11A^a^, T12A^a^, T13A^a^, T15A^a^, T20A^a^, T21A-ss-T3B^a^, and T20B-ss-T22B^a^ for abrin-a isoform (Table S1 marked in red). Seven specific peptides of T5A^b^, T10A^b^, T11A^b^, T12A^b^, T15A^b^, T16A^b^, and T22A-ss-T3B^b^ are unique to the abrin-b isoform (Table S2 marked in red). Notably, due to the extremely high amino acid sequence similarity (>92%) between abrin-c and abrin-d they have not individually specific peptides produced by trypsin digestion [18]. Therefore, eleven specific peptides shared by abrin-c and abrin-d isoforms were screened out including T5A^c,d^, T7A^c,d^, T8A^c,d^, T9A^c,d^, T10A^c,d^, T12A^c,d^, T13A^c,d^, T14A^c,d^, T15A^c,d^, T22A-ss-T2B^c,d^, T1B^c,d^, and T5B-ss-T7B^c,d^ (Tables S3 and S4 and marked in red).

**Figure 1:**
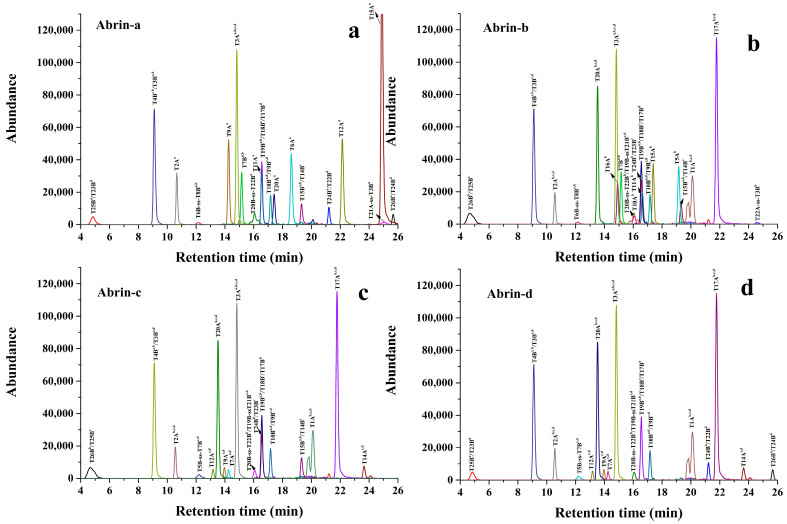
High-resolution liquid-mass extraction ion chromatograms of peptide markers from four isoforms of abrin. (**a**) High-resolution liquid-mass extraction ion chromatograms of peptide markers from abrin-a; (**b**) High-resolution liquid-mass extraction ion chromatograms of peptide markers from abrin-b; (**c**) High-resolution liquid-mass extraction ion chromatograms of peptide markers from abrin-c; (**d**) High-resolution liquid-mass extraction ion chromatograms of peptide markers from abrin-d.

To obtain marker peptides available for differential detection of abrin isoforms, abrin (AS172.9) was digested using the ACN-assisted trypsin digestion method we’d previously developed [17], the resulting sample was analyzed by LC-QTOF-MS. The specific peptide mapping of the four abrin isoforms was constructed by the extracted ion chromatogram (EIC) of the accurate mass of each abrin-specific peptide (Figure 1). As a result, for abrin-a (Figure 1a), except for the three peptides of T1A^a^, T13A^a^, and T7B^a,b^, all specific peptides were confirmed, their observed ions with the highest response were indicated bold (Table S1). These confirmed specific peptides with the highest MS response and good chromatography behavior are correspondingly selected as marker peptides. Based on these principles, a total of five abrin specific peptides of T2A^a^, T3A^a,b,c,d^, T6A^a^, T9A^a^, and T12A^a^ from A-chain, as well as three peptides of T4B^a,b^/T3B^c,d^, T10B^a,b^/T9B^c,d^, and T19B^a,b^/T18B^c^/T17B^d^ from B-chain are correspondingly selected as marker peptides (Table S5). Notably, T15A^a^ possessed a good MS response, but it was not selected as a marker peptide due to its strong hydrophobicity and the strong chromatographic retention in the C18 column. Among the selected marker peptides, the four peptides of T3A^a,b,c,d^, T4B^a,b^/T3B^c,d^, T10B^a,b^/T9B^c,d^, and T19B^a,b^/T18B^c^/T17B^d^ were regarded as the marker peptides for the identification of total abrin because of their common amino acid sequences for the four type of abrin isoforms. The four peptides of T2A^a^, T6A^a^, T9A^a^, and T12A^a^ were used as the marker peptides for the unambiguous identification of abrin-a isoform due to their uniqueness to abrin-a. Similarly, the three peptides of T15A^b^, T5A^b^, and T16A^b^ were selected as the marker peptides for the differential identification abrin-b isoform (Figure 1b and Table S6). The four peptides of T9A^c,d^, T12A^c,d^, T14A^c,d^, and T7A^c,d^ were screened as the marker peptides for the unambiguous identification of abrin-c/d (Figure 1c,d and Table S7). Finally, a total of 15 common or isoform-specific tryptic peptides were selected for the identification and quantification of abrin and its isoforms (Table S8). 

### 2.2. Enrichment and Purification of Abrin in Complex Matrix by Immunocapture

To remove background interferences in complex matrices and improve the method sensitivity, enrichment, and purification are vital procedures before trypsin digestion. In this work, we introduced a simple procedure of IMBs immunocapture to perform enrichment and purification. The IMBs were prepared according to our previously published method [15], in which biotinylated polyclonal antibody was coupled to streptavidin magnetic beads. The polyclonal antibody on the IMBs can recognize all isoforms of abrin (Supplementary information, Figure S1). It was found that the best enrichment efficiency was obtained under the condition of 1:2 of the mass ratio of a polyclonal antibody to biotin coupled with 18:250 of the mass ratio of a biotinylated polyclonal antibody to streptavidin magnetic beads. The maximum binding capacity of SA-MBs to biotinylated polyclonal antibody was 58.0 μg/mg using the BCA assay. There was the highest immunocapture recovery ratio of 75.4% when the 25 μL of abrin IMBs was added to 250 μL of 2.0 μg/mL abrin for enrichment. The affinity performance of IMBs were calculated to be about 15.08 μg of abrin per milligram, which was comparable with that of the previous study, suggesting that the developed immunocapture method was effective for the purification of abrin from complex matrices. 

### 2.3. Development of ACN- and Ultrasound-Assisted On-Bead Trypsin Digestion of Abrin

**Figure 2:**
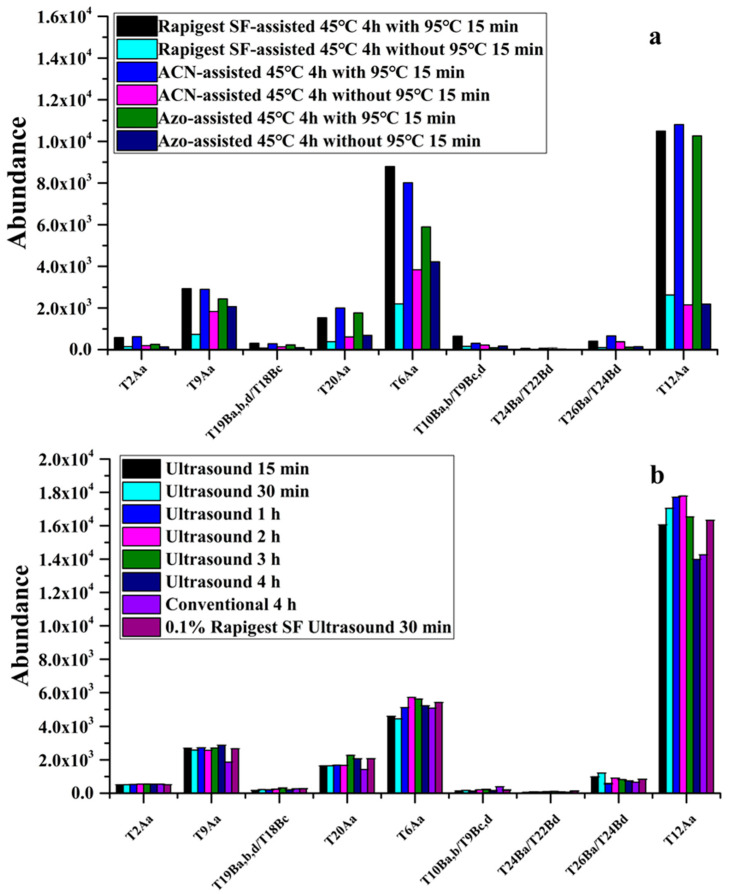
The influence of different denaturation conditions on the yields of abrin marker peptides (**a**) and the comparison of trypsin digestion protocols (**b**).

For the MS-based detection of protein toxins, trypsin digestion is the most time-consuming step, which normally takes up to 18–24 h [19]. To address these, some strategies were employed, such as immobilized trypsin [20], organic solvent assistance, surfactant-assisted protocols [21,22], hot acid digestion [23], microwave- [24], and ultrasound-assisted protocols [18]. As we know, protein denaturation and unfolding is a crucial step during digestion, especially for ricin and abrin that are resistant to proteases due to their compact structure. Surfactants of RapiGest SF and a photocleavable anionic surfactant [14,18], 4-hexylphenylazosulfonate (Azo) [22], ACN-Assisted trypsin digestion [17] were used to improve digestion efficiency by increasing protein solubilization and unfolding. To obtain the optimal protocols of solubilization and denaturation, we perform the protein denaturation experiment before the on-bead trypsin digestion. Based on previous studies, the three types of denaturant (10% ACN, 0.1% RapiGest SF and 0.1% Azo) are respectively used for denaturation of the captured abrin-a on IMBs during on-beads trypsin digestion for 4 h at 45 °C. Simultaneously, whether or not the factor of subjecting heating at 95 °C for 15 min before trypsin digestion are also considered. Each level of two factors was separately assessed against the yields of marker peptides of abrin-a (Figure 2a). The results indicate that performing the heating at 95 °C for 15 min before trypsin digestion had a significant effect on digestion efficiency, which contributed to improving the MS response of the marker peptides. The type of surfactant was critical for unfolding the intact abrin to increase marker peptide yields. The surfactants of 0.1% RapiGest SF and 10% ACN were optimal with the highest and comparable marker peptide yields, and the presence of 0.1% Azo exhibits a lower yield. Considering that RapiGest SF inhibits the ionization efficiency of ESI source and it was required for addition of HCl to degrade it for 45 min after digestion [18], resulting in sample preparation time prolonged, we chose 10% ACN as the digestion denaturant and heated at 95 °C for 15 min before trypsin digestion to promote protein unfolding.

Furthermore, to rapidly quantify abrin in complex matrices, ultrasound-assisted on-bead trypsin digestion conditions were fully optimized. Digestion temperature (25, 37, 45, and 55 °C) and incubation time (15 min, 30 min, 1 h, 2 h, 3 h, and 4 h) were respectively investigated against the yield of marker peptides of abrin-a. It was found that under the same digestion time, a maximum peptide yield was observed at 45 °C. It only takes 15 min to achieve a high and stable peptide yield under ultrasound-assisted trypsin digestion at 45 °C (Figure 2b). 

To evaluate this method performance, the optimized method was compared with the established method including our previous ACN-assisted digestion method [17] (45 °C for 4 h) and the method published by Hansbauer et al., where the ultrasound-assisted digestion was performed at 35 °C for 30 min, after digestion, appropriate HCl was added to degrade the excessive RapiGest SF for 45 min [18]. It turned out that our established ACN- and ultrasound-assisted digestion method gave comparable yields for all marker peptides of abrin-a with the other two methods (Figure 2b). In our developed method, ACN as denaturant was first introduced in the ultrasound-assisted on-bead trypsin digestion instead of traditional surfactant RapiGest SF, which significantly reduces the entire digestion time (covering protein denaturation and on-bead digestion) from 90 min [18] to 30 min. The built digestion method allows for the fast, effective and reproducible digestion of abrin into marker peptides suitable for further quantitative experiments.

### 2.4. HPLC-MS/MS (MRM) Method Development

To establish a sensitive HPLC-MS/MS (MRM) analysis method, the MRM parameters of the 15 abrin marker peptides including precursor-to-product transition ions, fragmentor, and collision energy were separately optimized using MassHunter software (Agilent). These optimized parameters of the precursor-to-product transition ions for all fifteen marker peptides and their corresponding isotope-labeled (R, label 13C_6_,15N_4_) synthetic peptides (AQUA for absolute quantification) are presented in Table 1. Among the 15 marker peptides, the precursor ions of three marker peptides of T12A^a^, T6A^b^, and T14A^c,d^ were dominated by the triply charges pseudo-molecular ions, and the rest twelve marker peptides by doubly charged pseudo-precursor ions. The marker peptide of T3A^a,b,c,d^ was chosen as the common quantitative peptide of all four abrin isoforms with one quantitative transition ion of 438.9 (2^+^)→429.6 based on the best MS response. The marker peptide of T12A^a^ was specially chosen as the quantitative peptide of abrin-a isoform with one quantitative transition ion of 676.2 (3^+^)→670.2, T15A^b^ as a quantitative peptide of abrin-b isoform with 619.0 (2^+^)→610.0, T9A^c,d^ as a quantitative peptide of abrin-c/d isoform with 514.8 (2^+^)→685.4. 

**Figure 3:**
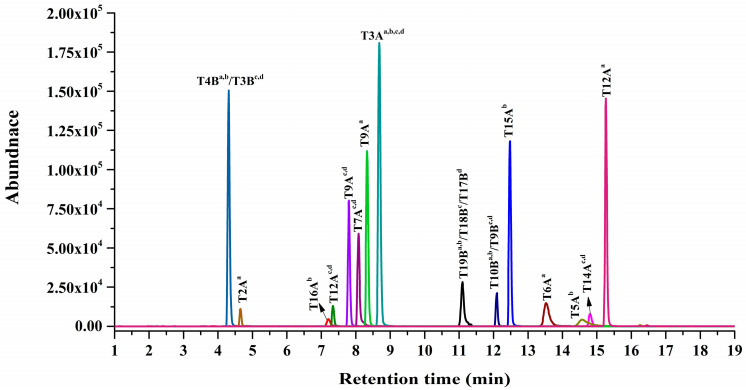
MRM chromatograms of all 15 abrin peptide markers.

To obtain good HPLC separation of 15 marker peptides from the plasma, parameters of chromatographic columns and elution program were evaluated. It was found that one Zorbax Eclipse Plus C_18_ reversed-phase analytical column (2.1 × 100 mm, 1.8 μm) was good separation with the column temperature of 30 °C. The chromatographic gradient separation conditions were carefully optimized. The optimized elution program contains four main isocratic segments, allowing for complete separation of the 15 marker peptides from the processed plasma sample, except for T15A^b^ and T14A ^c,d^ (Figure 3).

### 2.5. Method Validation

In response to quantify abrin in potential biological concern samples, the method performance was evaluated in milk and rabbit plasma matrices that respectively represent food and potential clinical matrices. The established method was evaluated on specificity, linearity, sensitivity, reproducibility, and stability.

#### 2.5.1. Specificity

The specificity of the method was determined by analyzing two matrix blank samples (milk and plasma) as well as 500 ng/mL of abrin’s homologous protein *A. precatorius* agglutinin in either milk or plasma. These samples were immunocaptured and ultrasound-assisted on-bead trypsin digestion and then analyzed by HPLC-MS/MS (MRM). The MRM transition ions of each marker peptide in matrix blank samples and *A. precatorius* agglutinin-containing sample were compared with that of a positive control sample, and the results showed that no background/interference peaks present for all the targeted abrin marker peptides, suggesting a good specificity of the method. 

#### 2.5.2. Linearity, Sensitivity, Accuracy and Precision (RSD)

Abrin standard (AS 172.9) was spiked into milk and plasma to prepare ten-point standard calibrator samples, ranging from 0.5–2000 ng/mL for total abrin, 0.235–940 ng/mL for abrin-a, 0.171–684 ng/mL for abrin-b, and 0.094–376 ng/mL for abrin-c/d. Each calibrator sample was subjected to immunocapture, ultrasound-assisted on-bead trypsin digestion, spiked with AQUA peptides, and analyzed by HPLC-MS/MS (MRM). The peptide of T3A^a,b,c,d^, T12A^a^, T15A^b^, and T9A^c,d^ were used for quantification of total abrin, abrin-a, abrin-b, and abrin-c/d, respectively. Calibration curves were respectively generated by linear regression analysis (Figure S1). The matrix-matched calibration curves showed good linearity in the range of 1.0–500 ng/mL for total abrin, 2.35–235 ng/mL for abrin-a, 3.42–513 ng/mL for abrin-b, and 9.40–376 ng/mL for abrin-c/d in milk and plasma, with a coefficient of determination value (R^2^) > 0.995. The limit of detection (LOD) values were calculated to be 0.5 ng/mL for T3A^a,b,c,d^, 0.47 ng/mL for T12A^a^, 1.71 ng/mL for T15A^b^, and 5.64 ng/mL for T9A^c,d^ ether in milk or plasma (Table 2). Furthermore, it was found that all quantification peptides could be detected at the concentration of total abrin of 30.0 ng/mL. T12A^a^, T15A^b^, and T9A^c,d^ with high MS response were novel peptides used for abrin quantification. The LODs of abrin-a and abrin-b in milk and plasma sample are lower than that of the published method (3.0 ng/mL) [18]. 

To assess intra-day/inter-day accuracy and precision, three replicates of quality control samples were measured at low, medium, and high concentrations (QCL, QCM, and QCH). As a result, the intra-day (*n* = 3) mean accuracy for abrin marker peptides was 86.7–107.4% in milk and 86.2–111.1% in plasma. Their corresponding intra-day precision values were ≤8.8% and ≤8.5%, respectively. The inter-day (*n* = 8) mean accuracy for all analytes were 83.5–94.7% in milk and 83.0–109.0% in plasma. Their corresponding inter-day precision values were ≤11.4% and ≤11.3%, respectively (Table 3). 

#### 2.5.3. Immunocapture Recovery and Matrix Effects

To evaluate immunocapture recovery of abrin from different matrices, we compared the content of total abrin in eluent with that in eluate during immunocapture at three levels (QCL, QCM, and QCH) in triplicate. The results showed that the immunocapture recovery of abrin was 71.1–76.3% in milk, and 73.4–80.9% in plasma matrix (Table 4). In addition, the matrix effects of the targeted marker peptides in milk and plasma were also investigated. The standard peptides of T3A^a,b,c,d^, T12A^a^, T15A^b^, and T9A^c,d^ were added to the processed matrix blank samples (milk and plasma) to a final concentration of 2.0. (QC-L), 20 (QC-M) and 100 (QC-H) ng/mL. The matrix-free samples were prepared at the same concentration in parallel. The matrix effect was calculated by comparing the MRM areas of each marker peptide between matrix-spiked and matrix-free samples at three spiking levels in triplicate. As a result, the mean matrix effects at three QC levels were 94.3–128.8% in milk and 91.9–125.9% in plasma (Table 5, Table S9).

#### 2.5.4. Stability

**Figure 4:**
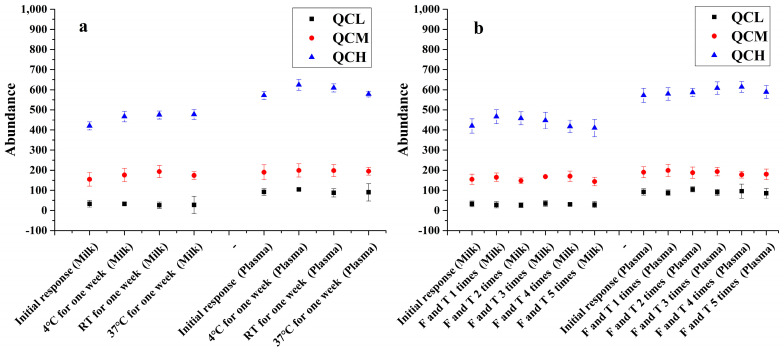
The investigation of sample stability. (**a**) The stability of the processed QC samples at 4 °C, room temperature and 37 °C for one week; (**b**) the stability of the QC samples after 5 times freeze-thaw cycles.

The temperature stability of marker peptides in processed samples and the freeze-thaw stability of abrin-containing samples were evaluated at the three QC levels with triplicates. The processed QC samples were stored at different temperatures (4 °C, room temperature, and 37 °C) for one week and then analyzed by HPLC-MS/MS (MRM). As a result, the targeted marker peptides from all QC samples were stable within ±15% variation of initial values at different temperatures for one week (Figure 4a). In addition, QC samples containing the abrin were frozen and thawed between −20 °C and 4 °C for 5 times before sample preparation and HPLC-MS/MS analysis. It was found that all QC samples were stable with the comparable area during the condition of freezing and thawing 5 times (Figure 4b). 

### 2.6. Method Application

#### 2.6.1. Characterization of the Purified Abrin from Gel Filtration Chromatography

To characterize abrin isoforms, the ACN-assisted trypsin digestion method combined with HPLC-MS/MS was applied to unambiguously detect abrin isoforms from different fraction samples of the four absorption peaks using gel filtration separation (from 32nd to 65th tube fraction sample, Figure S2). AQUA (T3A^a,b,c,d*^/T12A^a*^/T15A^b*^/T9A^c,d*^) peptides as ISTD were spiked into the samples for quantitation after trypsin digestion.

The relative moles of abrin-a, abrin-b, and abrin-c/d were calculated and represent the relative content of different abrin isoforms in purified abrin according to calculation equations described in Section 4.9. In the first absorption peak, the percentage of abrin-a showed a significant increasing trend from 23.1% to 54.2% with fraction numbers elevated from 32, 34, 35 to 37. The content of both abrin-b and abrin-c/d declined slowly from 44.9 to 28.6% and from 32.0 to 17.2%, respectively (Table S10). In the second absorption peak containing fraction number 40, 42, and 43, abrin-b is the major component, showing a significant increase percentage of abrin-b from 30.8% to 55.8%. The content of abrin-c/d exhibits a declining trend from 47.8 to 32.8%. The content of abrin-a was a minor component only accounting for 2.3–11.5% despite the content of abrin-a had an increasing trend. In the third absorption peak involving fraction number 53, 54, 55, and 57, only peptide markers of abrin-a were detected, indicating that abrin-a accounts for 100% of the total abrin. As for the fourth absorption peak covering fraction number 60, 61, and 65, its major component is also abrin-a with a percentage range of 81.3–77.0%. The low content of abrin-b is present ranging from 18.7 to 23%, and abrin-c/d peptide markers have not been detected. It was thought that abrin isoforms presented in different peaks, which might be caused by the sequence variation and the heterogeneous glycosylation modification on multisite. In addition, the same abrin isoform exists in different gel filtration chromatographic peaks. It is probably due to the difference in stereo structure and heterogeneous glycosylation of the same abrin isoform. In addition, according to previous reports [25], it would be helpful to elaborate on the content distribution of the four abrin isoforms from the perspective of transcription and genome levels.

Notably, the relative content of different abrin isoforms in the abrin standard sample (AS 172.9) provided by OPCW in the third biotoxin analysis exercise was also determined. The results showed that abrin-a accounts for 47%, and abrin-b for 34.2%, abrin-c/d for 18.8% of the total abrin, which of relative content is identical to that of the fraction number 35 and 37 in the first adsorption peak. Similar relative content was also found by Hansbauer E M et al. [18], who verified that the main component was abrin-a and minor was abrin-b and abrin-c/-d in *Abrus* seeds. In current work, trypsin digestion combined with MRM targeted detection was used for simultaneous identification of marker peptides from all abrin isoforms, which allowed the accurate determination of the relative content of abrin isoforms in purified abrin. 

#### 2.6.2. Identification of Abrin from the Samples of Biotoxin Exercises

The developed method was applied for the unambiguous identification of abrin from the unknown samples of the second, third and fourth biotoxin exercises organized by OPCW. The identification criteria of abrin were performed according to the guidelines [26] of the OPCW biotoxin exercise, in which at least two marker peptides from each chain of abrin must be recorded with at least three MRM transitions corresponding to each marker peptide. The developed sample preparation and HPLC-MS/MS (MRM) method were applied to all samples to identify abrin, the results were shown in Table 6. In the samples of the second biotoxin exercise, three solid samples were pretreated for extraction of abrin from Stevia powder using three to ten volumes of 2% acetic acid. The aqueous extract of solid samples and four complex aqueous samples were then processed with immunocapture purification and the on-bead trypsin digestion described in Section 4.4 and analyzed using HPLC-MS/MS (MRM) described in Section 4.7. As a result, intact abrin of two positive samples spiked with purified abrin (A172.4 and A172.5) were unambiguously identified. All twelve abrin marker peptides from A-chain (T3A^a,b,c,d^, T12A^a^, T9A^a^, T6A^a^, T2A^a^, T15A^b^, T5A^b^, T16A^b^, T9A^c,d^, T7A^c,d^, T12A^c,d^, and T14A^c,d^) and 3 marker peptides from B chain (T4B^a,b^/T3B^c,d^, T19B^a,b^/T18^c^/T17B^d^, and T10B^a,b^/T9B^c,d^) were clearly identified according to the guidelines of OPCW biotoxin exercise for accurate identification of abrin (Table 6). In the samples of the third biotoxin exercise, five powder samples (BT18.1-BT18.5) were pretreated for extraction of abrin using 2% acetic acid and then ammonium sulfate precipitation. The aqueous extract of protein powder samples and complex aqueous samples were then processed and analyzed with the same procedure described in Section 4.4 and Section 4.7. As a result, intact abrin of two positive samples spiked with purified abrin (BT18.1 and BT18.2) were unambiguously identified. In these samples, eight marker peptides of TT3A^a,b,c,d^, T12A^a^, T9A^a^, T6A^a^, T2A^a^, T15A^b^, T9A^c,d^ and T12A^c,d^ in A-chain and three marker peptides of T4B^a,b^/T3B^c,d^, T19B^a,b^/T18^c^/T17B^d^ and T10B^a,b^/T9B^c,d^ in B chain were clearly identified (Table 6). In the samples of the fourth biotoxin exercise, three SDS-PAGE gel samples were pretreated for transferring the toxin proteins to the aqueous solution using electroelution. The electroeluted solution and the four aqueous samples were prepared and analyzed. As a result, intact abrin was unambiguously identified in the positive sample spiked with purified abrin (BT19.3). Six marker peptides of T3A^a,b,c,d^, T12A^a^, T9A^a^, T6A^a^, T2A^a^ and T15A^b^ in A-chain and three marker peptides of T4B^a,b^/T3B^c,d^, T19B^a,b^/T18^c^/T17B^d^ and T10B^a,b^/T9B^c,d^ in B chain were clearly identified (Table 6). All the results of the above analysis meet the stringent requirements for accurate identification of abrin for the evaluation of OPCW biotoxin exercise and forensic analysis related to the Chemical Weapons Convention.

## 3. Conclusions

This study provides a rapid and differential quantification method for abrin and its isoforms in complex food and clinical matrices. Identification and selection of the marker peptides of abrin isoforms by in silico digestion and BLASTp database search combined with HPLC-HRMS analysis. Fifteen specific tryptic peptides with a good mass response and chromatographic behaviors were selected as marker peptides for the identification and quantification of abrin and its isoforms. Among them, T3A^a,b,c,d^, T12A^a^, T15A^b^, and T9A^c,d^ were considered as the quantitative peptide for total abrin, abrin-a, abrin-b, and abrin-c/d, respectively. The immunocapture was used to extract abrin from complex matrices effectively. One ultrasound-enhanced trypsin digestion method combined with the acetonitrile-assisted digestion strategy was established for achieving efficient and rapid trypsin digestion. ACN as a denaturant instead of the traditional surfactant RapiGest SF was first introduced in the ultrasound-assisted on-bead trypsin digestion of abrin. The cleavage efficiency was significantly increased and the entire digestion time was shortened from 90 min to 30 min. Furthermore, quantification was performed using isotope-labeled tryptic peptides as IS and HPLC-MS/MS operating in MRM mode. The limit of quantification of abrin was down to 1.0 ng/mL in milk and plasma. The method was fully validated and applied for the characterization of abrin and its isoforms from various fractions during fine purification using gel filtration separation. Furthermore, the method was successfully used for the unambiguous identification of abrin in the samples of the second, third, and fourth biotoxin exercises organized by OPCW. The study provides a rapid and sensitive method, which is suitable for promotion in international laboratories to improve the identification capability of abrin in complex matrices.

## 4. Materials and Methods

### 4.1. Safety Precaution

Abrin is a highly toxic protein belonging to type II ribosome-inactivating proteins, which would infect humans via ingestion, inhalation, or exposure through broken skin. All abrin-related experiments should be performed in a biosafety level-2 cabinet equipped with a HEPA filter. Abrin contaminated solutions and consumables must be decontaminated with 2 M NaOH.

### 4.2. Chemicals and Reagents

Acetonitrile (HPLC grade), water (HPLC grade), and formic acid (>98%) were purchased from Honeywell Burdick & Jackson (Muskegon, MI, USA). Sodium chloride (NaCl), sodium dihydrogen phosphate (NaH_2_PO_4_), disodium hydrogen phosphate (Na_2_HPO_4_), ammonium chloride (NH_4_Cl), sodium hydrogen carbonate (NaHCO_3_), ammonium hydrogen carbonate (NH_4_HCO_3_), were purchased from Sigma-Aldrich Co. (St. Louis, MO, USA). Biotin N-hydroxy succinimide esters (NHS-Biotin) were purchased from Sigma-Aldrich Co. (St. Louis, MO, USA). Streptavidin MagneSphere Paramagnetic Particles (SA-magnetic beads, MBs suspension of 1 mg of MBs/mL) and trypsin (Modi seq) were purchased from Promega (Madison, WI, USA). RapiGest SF Surfactant was purchased from Waters Corporation (Milford, MA, USA). The photocleavable anionic surfactant, 4-hexylphenylazosulfonate (Azo), was synthesized in our laboratory according to Kyle B A’s method [22]. The anti-abrin polyclonal antibody (pAb) was kindly donated by Professor Zhao-Yang Tong from the State Key Laboratory of NBC Protection for Civilian. Centricon centrifugal filter device with a molecular weight cutoff of 10 kDa was purchased from Merck Millipore (Darmstadt, Germany). Plasma matrix from rabbit was donated from State Key Laboratory of NBC Protection for Civilian.

The isotope-labeled peptides of T12A^a*^ (QQIPLGLQALTHGISFF[^13^C_6_;^15^N_4_]R), T15A^b*^ (QQIPLGLQA L[^13^C_6_;^15^N_4_]R), T9A^c,d*^ (AGSQSYFL[^13^C_6_;^15^N_4_]R) and T3A^a,b,c,d*^ (QFIEAL[^13^C_6_;^15^N_4_]R) were purchased from Synpeptide Co., Ltd. (Nanjing, China). The purified abrin (AS172.9) which contains all isoforms was obtained from the OPCW laboratory by participating in the Biotoxin Sample Analysis Exercise. 

### 4.3. Samples for Calibration and Quality Control

#### 4.3.1. Milk and Plasma Samples for Calibration and Quality Control

A series of abrin calibration standards were prepared by stepwise dilution of 100 μg/mL abrin (AS172.9) stock solution with milk and plasma to a final concentration of 2000, 1500, 1000, 500, 100, 50.0, 30.0, 5.00, 1.00, and 0.50 ng/mL. Similarly, the quality control (QC) samples were prepared to a final concentration of 10.0 ng/mL (QCL), 150 ng/mL (QCM), and 300 ng/mL (QCH) by serial dilution of 1.0 μg/mL abrin (AS172.9) with milk and plasma, respectively. 

The matrix-spiking samples were prepared by spiking three levels of T3A^a,b,c,d^, T12A^a^, T15A^b^, and T9A^c,d^ in the processed blank milk and plasma samples with a concentration of 2.00 ng/mL (QC-L), 20.0 ng/mL (QC-M), and 100 ng/mL (QC-H). Peptides of T3A^a,b,c,d*^, T12A^a*^, T15A^b*^and T9A^c,d*^were spiked in H_2_O to prepare the AQUA internal standard peptides (1.0 μg/mL). Calibrators, QC samples, matrix-spiking samples, and internal standards were stored at 4 °C until use.

#### 4.3.2. Abrin Extracts and Fraction Samples from the Fine Purification

According to the previous method described with slightly modified, abrin was purified from *A. precatorius* seeds from Xishuangbanna, Yunnan Province, China [4]. Briefly, peeled seeds were extracted with 2% acetic acid. The total proteins from extracts were precipitated with 80% ammonium sulfate and then dissolved in 1 × PBS (2 mM NaH_2_PO_4_, 8 mM Na_2_HPO_4_, 140 mM NaCl, pH 7.2) solution to obtain crude abrin extracts. Affinity chromatography was employed to isolate abrin isoforms and agglutinin using a Galactose-Sepharose resin pre-packed column. Abrin isoforms and agglutinin were continued purified by anion exchange chromatography on a DEAE-Sepharose FF column. Each component of anion exchange was checked by SDS-PAGE, and the abrin component with a molecular weight of about 63 kD is subjected to gel filtration with a packed Sephacryl S-200 column. Each fraction from the gel filtration separation was collected and its concentration was determined by BCA assays. The collected samples were stored at 4 °C until HPLC-MS/MS analysis.

### 4.4. Immunocapture and Ultrasound-Assisted on-Bead Trypsin Digestion

The immunomagnetic beads (IMBs) were prepared by coupling 72 µg biotinylated polyclonal abrin antibody to 1.0 mg of streptavidin-coupled magnetic beads (SA-MBs) according to our previously developed method [15]. Abrin-containing samples (250 μL) were incubated with 25 μL of the prepared IMBs for 1 h with gentle shaking. The IMBs were resuspended in digestion buffer (77 μL of H_2_O, 10 μL of ACN, and 5 μL of 1 M NH_4_HCO_3_) following the sequential washing with 1.0 mL of PBST, 500 μL of PBS, and 500 μL of distilled water. The resuspended IMBs were denatured at 95 °C for 15 min, and an 8 μL volume of 0.25 mg/mL trypsin was added after cooling to room temperature. The IMBs samples were digested at 45 °C for 15 min in an ultrasound water bath. The reaction was terminated by adding 10% formic acid with pH < 4. The samples were centrifuged at 13000 rpm for 10 min. The supernatant was transferred to vials and added with 2 μL of 1.0 μg/mL AQUA peptides before HPLC-MS/MS analysis. 

### 4.5. In Silico Digestion and Uniqueness Identification of Digested Peptides 

The amino acid sequence of abrin-a, -b, -c, and -d (P11140, Q06077, P28590, and Q06076) was downloaded from the UniProt database (https://www.uniprot.org/ (accessed on 10 February 2021). The reported sequence alignment shows that the sequence similarity of the four isoforms is as high as 78%, (the sequence homology of abrin-c and abrin-d is about 92.3%) [18]. In silico digestion of abrin into tryptic peptides were generated using ExPASy peptide mass tool (https://web.expasy.org/peptidemass/ (accessed on 13 February 2021). The uniqueness of the theoretical digested peptides was identified by NCBI BLASTp (https://blast.ncbi.nlm.nih.gov/Blast.cgi (accessed on 13 February 2021) searching against the non-redundant protein database (date of database access: 13 February 2021). 

### 4.6. LC-QTOF MS Analysis for Identification

High-resolution LC/MS data were acquired using an Agilent 1200 HPLC system combined with a 6520 high-resolution accurate quadrupole-time-of-flight (QTOF) mass spectrometer with a mass resolution of 10,000. Ten microliters of tryptic samples were separated on an Advanced Peptide Map 2.7-μm, 2.1 × 150 mm column (Agilent technology, Palo Alto, MA, USA). Mobile phase A consisted of 0.1% formic acid in water. Mobile phase B consisted of 0.1% formic acid in acetonitrile. The flow rate and the column temperature were respectively 0.25 mL/min and 30 °C. The gradient elution program is as follows: 5%B held at 0 to 3 min, a linear gradient was applied ramping from 5% to 70% between 3 and 42 min, 42–50 min 70%B; the content of B% was increased to 100% within 1 min and held for 8 min before reverting to the initial 5%B. The eluted peptides were injected into an Agilent 6520 QTOF mass spectrometer. The scan range was 150–3000 at 1.1 spectra/s; the gas temperature was 330 °C, the gas flow was 8 L/min, nebulizer voltage was 30 psi, and capillary voltage was 4000 V; Fragmentor was 150 V and skimmer was 65 V. 

### 4.7. HPLC-ESI MS/MS Analysis for Quantification

For abrin quantification by targeted MS (MRM), the analysis was performed using an Agilent Technologies 6460 triple quadrupole mass spectrometer equipped with a 1290 Affinity series HPLC system. Chromatographic separation was performed on a C_18_ guard column (2.1 × 5.0 mm, 1.8 μm) and a Zorbax Eclipse Plus C_18_ reversed-phase analytical column (2.1 × 100 mm, 1.8 μm). Mobile phase A consisted of 0.1% formic acid in water. Mobile phase B consisted of 0.1% formic acid in acetonitrile. The flow rate for analysis was 0.3 mL/min and the column temperature was 30 °C. The optimized gradient program was: 5%B held at 0 to 1.8 min, mobile phase B was then linearly ramped to 14% within 0.2 min and held for 2.5 min, subsequently ramped to 19% within 0.5 min and held for 4.0 min, raised to 25% within 0.2 min and hold for 3.8 min, raised to 45% within 0.2 min and hold for 3 min, sharply increased to 100% within 0.3 min and held for 4.5 min. The column was finally re-equilibrated with 5% mobile phase B for 4.0 min before the next run. Eluted peptides were introduced to the 6460 triple quadrupoles mass analyzer fitted with an ESI ionization source operating in positive ionization and MRM mode. The injection volume was 10 μL.

The gas temperature was 350 °C, the flow rate of gas was 10 L/min, 35 Psi of nebulizer gas, and 4000 V of capillary voltage. The confirmation and quantification of each marker peptide were performed by recording three diagnostic precursor-product ion MRM transitions. For each transition, the MS/MS acquisition parameters for collision energy, fragmentor, and cell accelerator voltage were independently optimized and showed in Table 1.

### 4.8. Method Validation

Milk (purchased from a local market) and rabbit plasma were filtered with 0.45 μm membrane before use. A 10-point standard curve was generated for milk and plasma between 0.5 and 2000 ng/mL of total abrin (corresponding to 0.47–940 ng/mL of abrin-a, 0.342–684 ng/mL of abrin-b, 0.188–376 ng/mL of abrin-c/d). Total abrin concertation of 10.0, 150, and 300 ng/mL was served as QC samples. One complete analytical run consisted of a matrix blank sample, three QC samples (QCL, QCM, and QCH), and a complete ten-point calibration standard. 

For specificity evaluation, matrix blank samples (milk and plasma) and 500 ng/mL of *Abrus* agglutinin spiked milk and plasma were analyzed using the established method to confirm the background/interference peaks for each marker peptide of abrin. 

Linear regression was applied to generate a calibration curve. The LOD was defined as three times the signal-to-noise ratio (S/N). The lower limit of quantification (LOQ) was defined as S/N > 10. The accuracy is calculated by dividing the abrin concentration measured from the abrin spiked sample by the original abrin spiked concentration. The intra-day/inter-day precision (%RSD) of QC samples should be below 20% for a set of measurements. The percent accuracy should be between 80% and 120% of the spiked value. The immunocapture recovery was calculated based on the T3A^a,b,c,d^ response ratio of abrin bound to IMBs to the combination of abrin in the effluent, washing solution, and the abrin bound to IMBs. The matrix effect was calculated by dividing the MS response of T3A^a,b,c,d^, T12A^a^, T15A^b^, and T9A^c,d^ in the matrix-spiked QC samples by that in the matrix-free samples. The stability was performed by determining the influence of temperatures (4 °C, room temperature and 37 °C) and freeze-thaw cycles (*n* = 5) on the response of T3A^a,b,c,d^, T12A^a^, T15A^b^, and T9A^c,d^ in three QC samples in triplicate.

### 4.9. Calculation of Relative Content of Different Abrin Isoforms 

Assuming that the trypsin digestion efficiency is 100% and similar for all abrin isoforms, the relative content of different abrin isoforms was achieved in the gel-filtration material using the peak area ratios of native peptides to AQUA peptide based on the calculated relative moles of different abrin isoforms according to calculation equations (Equations (1) and (2)).

(1) $$\{\begin{array}{c} a=\frac{A\left(T12A^{a}\right)}{A\left(T12A^{a*}\right)}/M\left(T12A^{a}\right)\\ b=\frac{A\left(T15A^{b}\right)}{A\left(T15A^{b*}\right)}/M\left(T15A^{b}\right)\\ c=\frac{A\left(T9A^{c,d}\right)}{A\left(T9A^{c,d*}\right)}/M\left(T9A^{c,d}\right) \end{array}$$

In the Equation (1), a, b, c represents the relative moles of abrin-a, abrin-b and abrin-c/d, respectively, A(T12A^a^) is the peak area of the peptide T12A^a^, A(T12A^a*^) is the peak area of the internal standard T12A^a*^. Similarly, A(T15A^a^), A(T9A^c,d^), A(T15A^a*^) and A(T9A^c,d*^) represents the peak area of peptide T15A^a^, T9A^c,d^ and their corresponding internal standard peptide T15A^a*^, T9A^c,d*^, respectively. M(T12A^a^), M(T15A^b^) and M(T9A^c,d^) represents the relative molecular weight of the peptide T12A^a^, T15A^b^ and T9A^c,d^, respectively. M(T12A^a^) = 2026.1232, M(T15A^b^) = 1236.7423, M(T9A^c,d^) = 1028.5160.

(2) $$\{\begin{array}{c} x=\frac{a}{a+b+c}*100\%\\ y=\frac{b}{a+b+c}*100\%\\ z=\frac{c}{a+b+c}*100\% \end{array}$$

In Equation (2), x, y, and z represent the relative percentage of abrin-a, abrin-b, and abrin-c/d in different gel filtration fractions of abrin.

## Figures and Tables

**Table 1:** The optimized MRM parameters for the HPLC-MS/MS analysis of marker and IS peptides.

Peptide	Amino Acid Sequences	Precursor Ion (m/z)	Product Ions (m/z)	Fragmentor (V)	Collision Energy (V)
T3A^a,b,c,d^	QFIEALR	438.9 (2+)	601.4, 488.7, 429.6	90	15, 10, 8
T2A^a^	FSTEGATSQSYK	653.4 (2+)	841.4, 713.3, 527.3	170	20, 20, 20
T6A^a^	GGLIHDIPVLPDPTTLQER	691.2 (3+)	1015.5, 902.5, 844.4	120	15, 20, 25
T9A^a^	AGTQSYFLR	521.8 (2+)	685.4, 598.3, 512.8	120	20, 10, 10
T12A^a^	QQIPLGLQALTHGISFFR	676.2 (3+)	829.4, 670.2, 553.4	160	15, 15, 15
T5A^b^	LTGGLIHGIPVLPDPTTLQER	743.2 (3+)	862.5, 844.4, 528.8	120	25, 30, 15
T15A^b^	QQIPLGLQALR	619.0 (2+)	867.6, 610.0, 257.2	140	20, 15, 20
T16A^b^	HAISFLQSGTDDQEIAR	630.2 (3+)	1091.6, 556.4, 86.2	130	15, 20, 30
T7A^c,d^	YITVELSNSER	656.0 (2+)	1034.6, 834.5, 249.2	150	20, 20, 25
T9A^c,d^	AGSQSYFLR	514.8 (2+)	685.4, 505.8, 441.8	110	15, 10, 15
T12A^c,d^	FDGSYGDLER	579.8 (2+)	896.8, 752.4, 589.4	130	20, 20, 20
T14A^c,d^	EEISLGLQALTHAISFLR	666.9 (3+)	814.1, 770.6, 714.4	140	15, 15, 15
T4B^a,b^/T3B^c,d^	YEPTVR	382.8 (2+)	472.3, 265.2, 236.7	90	8, 10, 8
T10B^a,b^/T9B^c,d^	LEENQLWTLK	637.4 (2+)	361.2, 243.1, 215.1	160	20, 20, 25
T19B^a,b^/T18B^c^/T17B^d^	EQQWALYTDGSIR	783.9 (2+)	924.4, 811.4, 368.2	170	25, 25, 30
T3A^a,b,c,d^*	QFIEALR*	443.9 (2+)	611.4, 498.3, 434.6	90	15, 12, 8
T12A ^a^*	QQIPLGLQALTHGISFFR*	679.5 (3+)	834.0, 673.5, 556.4	150	18, 15, 15
T15A ^b^*	QQIPLGLQALR*	624.0 (2+)	877.5, 614.9, 439.3	120	35, 15, 15
T9A ^c,d^*	AGSQSYFLR*	519.9 (2+)	695.4, 510.9, 446.8	100	15, 812

R*: [^13^C_6_;^15^N_4_] labeled arginine.

**Table 2:** Standard curve, linear range, limit of detection, and limit of quantification of different abrin isoforms in milk and plasma.

Peptide	Matrices	Calibration Curve	R^2^	LOD (ng/mL)	LOQ (ng/mL)	Range (ng/mL)
T3A^a,b,c,d^	Milk	Y = 0.3103X + 1.4980	0.998	0.50	1.00	1.00–500
T12A^a^	Milk	Y = 0.0851X − 0.1942	0.998	0.47	2.35	2.35–235
T15A^b^	Milk	Y = 0.0041X + 0.2192	0.996	1.71	3.42	3.42–513
T9A^c,d^	Milk	Y = 0.0041X + 0.4499	0.998	5.64	9.40	9.40–376
T3A^a,b,c,d^	Plasma	Y = 0.037X + 1.6101	0.999	0.50	1.00	1.00–500
T12A^a^	Plasma	Y = 0.0879X + 0.4292	0.999	0.47	2.35	2.35–235
T15A^b^	Plasma	Y = 0.0045X + 0.2651	0.996	1.71	3.42	3.42–513
T9A^c,d^	Plasma	Y = 0.0045X + 0.4597	0.995	5.64	9.40	9.40–376

**Table 3:** Intra/Inter-day accuracy and precision (RSD%) over all QC samples.

Sample	Quantitative Peptides	Matrices	Spiking Concentration (ng/mL)	Intra-Day (*n* = 3)			Inter-Day (*n* = 8)		
				Calculated Concentration (ng/mL)	Accuracy (%)	RSD (%)	Calculated Concentration (ng/mL)	Accuracy (%)	RSD (%)
QCL	T3A^a,b,c,d^	Milk	10.0 (total abrin)	9.76 ± 0.14	97.6	6.3	9.45 ± 0.19	94.5	11.4
QCL	T12A^a^	Milk	4.70 (abrin-a)	4.38 ± 0.04	94.7	8.8	4.07 ± 0.10	86.5	7.5
QCL	T15A^b^	Milk	3.42 (abrin-b)	3.67 ± 0.02	107.4	7.6	3.18 ± 0.16	93.1	7.8
QCL	T9A^c,d^	Milk	1.88 (abrin-c/d)	-	-	-	-	-	-
QCM	T3A^a,b,c,d^	Milk	150 (total abrin)	150.2 ± 3.90	100.1	3.7	142.0 ± 0.81	94.7	9.2
QCM	T12A^a^	Milk	70.5 (abrin-a)	67.4 ± 0.02	95.6	3.3	64.9 ± 0.11	92.1	8.2
QCM	T15A^b^	Milk	51.3 (abrin-b)	48.5 ± 1.04	94.6	2.7	46.1 ± 0.15	88.9	6.9
QCM	T9A^c,d^	Milk	28.2 (abrin-c/d)	24.4 ± 0.04	86.7	2.9	24.3 ± 0.08	86.1	4.9
QCH	T3A^a,b,c,d^	Milk	300 (total abrin)	288.0 ± 0.18	96.0	1.7	279.5 ± 8.71	93.2	5.5
QCH	T12A^a^	Milk	141 (abrin-a)	129.9 ± 0.95	92.1	2.9	123.1 ± 6.32	87.3	5.3
QCH	T15A^b^	Milk	102.6 (abrin-b)	97.8 ± 1.23	95.3	2.2	92.1 ± 4.83	89.8	2.9
QCH	T9A^c,d^	Milk	56.4 (abrin-c/d)	49.2 ± 1.74	87.2	3.8	47.1 ± 5.71	83.5	4.5
QCL	T3A^a,b,c,d^	Plasma	10.0 (total abrin)	11.2 ± 0.18	112.0	8.5	10.9 ± 0.23	109.0	9.9
QCL	T12A^a^	Plasma	4.70 (abrin-a)	6.88 ± 1.01	102.7	5.3	6.32 ± 0.88	94.0	8.8
QCL	T15A^b^	Plasma	3.42 (abrin-b)	3.02 ± 0.53	111.1	4.9	2.50 ± 0.58	92.9	11.3
QCL	T9A^c,d^	Plasma	1.88 (abrin-c/d)	-	-	-	-	-	-
QCM	T3A^a,b,c,d^	Plasma	150 (total abrin)	148.6 ± 3.3	99.1	3.2	142.6 ± 4.8	94.9	6.9
QCM	T12A^a^	Plasma	70.5 (abrin-a)	67.8 ± 5.6	96.2	4.8	64.9 ± 4.6	92.0	8.6
QCM	T15A^b^	Plasma	51.3 (abrin-b)	48.9 ± 1.8	95.3	2.9	42.6 ± 2.1	83.0	6.2
QCM	T9A^c,d^	Plasma	28.2 (abrin-c/d)	24.3 ± 0.8	86.2	5.6	24.9 ± 1.9	88.3	8.9
QCH	T3A^a,b,c,d^	Plasma	300 (total abrin)	293.3 ± 1.92	97.8	1.8	289.5 ± 2.3	96.5	4.4
QCH	T12A^a^	Plasma	141 (abrin-a)	132.4 ± 4.13	93.9	1.7	126.1 ± 5.3	89.4	5.3
QCH	T15A^b^	Plasma	102.6 (abrin-b)	98.7 ± 3.2	96.2	2.8	94.7 ± 3.3	92.3	5.9
QCH	T9A^c,d^	Plasma	56.4 (abrin-c/d)	53.2 ± 1.2	94.3	6.1	51.8 ± 2.5	91.8	8.6

**Table 4:** Immunocapture extraction recovery of abrin in milk and plasma.

Sample ID	Spiking Concentration (ng/mL)	Matrices	Extraction Recovery %	RSD %
QCL	10.0	Milk	76.3	3.3
QCM	150	Milk	77.4	2.1
QCH	300	Milk	71.1	1.8
QCL	10.0	Plasma	80.9	4.9
QCM	150	Plasma	77.8	2.8
QCH	300	Plasma	73.4	1.2

**Table 5:** Matrix effects of abrin peptide markers in milk and plasma.

Sample ID	Matrices	Spiking Concertation of Peptides (ng/mL)	Matrices Effect of T3A^a,b,c,d^ %	Matrix Effect of T12A^a^ %	Matrix Effect of T15A^b^ %	Matrix Effect of T9A^c,d^ %
QC-L	Milk	2.00	128.8 ± 2.8	110.8 ± 3.7	124.2 ± 5.6	106.9 ± 3.3
QC-M	Milk	20.0	106.2 ± 4.2	96.1 ± 4.1	96.5 ± 4.8	91.5 ± 2.8
QC-H	Milk	100	103.3 ± 1.1	94.3 ± 6.2	96.9 ± 3.1	102.7 ± 2.6
QC-L	Plasma	2.00	112.5 ± 2.6	113.0 ± 2.9	125.9 ± 2.5	110.9 ± 3.4
QC-M	Plasma	20.0	112.7 ± 3.6	97.3 ± 4.5	108.5 ± 2.6	98.1 ± 5.6
QC-H	Plasma	100	91.9 ± 2.9	92.8 ± 2.3	98.6 ± 3.7	96.5 ± 2.7

**Table 6:** The analysis results of the biotoxin sample analysis exercise.

Sample	Spiking Chemicals/(Nominal Concentration)	Matrices	Identification	Identified Peptide Markers
A172.1	Blank	BSA 1 mg/mL in 5% AcOH	-	
A172.4	Purified abrin (100 μg/mL)	BSA 1 mg/mL in 5% AcOH	Abrin	15 (12 from A chain and 3 from B chain)
A172.5	Purified ricin (100 μg/g) & purified abrin (1 mg/g)	Commercial Stevia powder	Abrin	15 (12 from A chain and 3 from B chain)
BT18.1	Purified * Abrin (150 μg/g)	Protein powder	Abrin	11 (8 from A chain and 3 from B chain)
BT18.2	Abrus Agglutinin * (300 μg/g) Purified Viscumin (50 μg/g) Purified Ricin (15 μg/g)	Protein powder	Abrin	11 (8 from A chain and 3 from B chain)
BT18.4	Blank	Protein powder	-	
BT18.7	Blank	Saline solution	-	
BT19.1	Blank (Contains BSA)	SDS-PAGE gel *		
BT19.3	Crude Castor Bean Extract (15 μg/spot) Purified Abrin (15 μg/spot)	SDS-PAGE gel	Abrin	9 (6 from A chain and 3 from B chain)
BT19.5	Blank	Spray buffer †	-	

* SDS-PAGE gel spot size was standardized by using a gel cutting tip. Gel pieces were dried before storage and shipping. † Spray buffer consists of PBST buffer (1000 mL), Polypropylene glycol monobutylether (120 mL), PEG200 (60 mL), Glycerol (50 mL), ethanol (100 mL) and isopropanol (100 mL). In the second biotoxin exercise, four aqueous samples (A172.1–A172.4, prepared in 1 mg/mL of BSA in 5% AcOH) and three solid samples (A172.5–A172.7, prepared in Stevia powder) were provided by the OPCW center laboratory. For solid samples, three to ten volumes of 2% acetic acid were used to extracted abrin from Stevia powder. In the third biotoxin exercise, five powder samples (BT18.1-BT18.5, prepared in protein powder) and two aqueous samples (BT18.6 and BT18.7, prepared in saline solution). In the fourth biotoxin exercise, three SDS-PAGE gel samples (BT19.1–BT18.3) and four aqueous samples (BT18.6 and BT18.7, prepared in spray buffer) were provided by the OPCW center laboratory.

## Data Availability

Data is contained within the manuscript or the supplementary information.

## Supplementary Materials

The following are available online at https://www.mdpi.com/article/10.3390/toxins13050358/s1, Figure S1: Standard curves of four type abrin peptide markers in milk (black) and plasma (red). Figure S2: Separation and purification of four isoforms of abrin by gel filtration chromatography. Table S1: Specific peptides of abrin-a generated by trypsin digestion. Table S2: Specific peptides of abrin-b generated by trypsin digestion. Table S3: Specific peptides of abrin-c generated by trypsin digestion. Table S4: Specific peptides of abrin-d generated by trypsin digestion. Table S5: Abrin-a specific peptides with high mass response produced by trypsin digestion. Table S6: Abrin-b specific peptides with high mass response produced by trypsin digestion. Table S7: Abrin-c/d specific peptides with high mass response produced by trypsin digestion. Table S8: Common or specific marker peptides of four abrin isoforms. Table S9: The relative content of different isoforms of abrin in the gel filtration fraction samples and abrin standard provided by OPCW. Table S10: The average MRM area (*n* = 3) values of the peptides in the matrix and matrix-free samples.
