# Supplementary material for: Rapid Differential Detection of Abrin Isoforms by an Acetonitrile- and Ultrasound-Assisted On-Bead Trypsin Digestion Coupled with LC-MS/MS Analysis
Source: Toxins (Basel). 2021 May 18;13(5):358. doi: 10.3390/toxins13050358 (PMC8157574; doi:10.3390/toxins13050358)
Supplement: Supplementary file 1 [file toxins-13-00358-s001.zip › toxins-1200502-supplementary.pdf]

Rapid Differential Detection of Abrin Isoforms by an Acetonitrile- and Ultrasound-Assisted On-Bead Trypsin Digestion Coupled with LC-MS/MS Analysis

Long-Hui Liang, Yang Yang, Shu Geng, Xi Cheng, Hui-Lan Yu, Chang-Cai Liu and Shi-Lei Liu

Contents of Appendix A. Supplementary data

Figure S1: Standard curves of four type abrin peptide markers in milk (black) and plasma (red).

Table S1: Specific peptides of abrin-a generated by trypsin digestion

Table S2: Specific peptides of abrin-b generated by trypsin digestion

Table S3: Specific peptides of abrin-c generated by trypsin digestion

Table S4: Specific peptides of abrin-d generated by trypsin digestion

Table S5: Abrin-a specific peptides with high mass response produced by trypsin digestion

Table S6: Abrin-b specific peptides with high mass response produced by trypsin digestion

Table S7: Abrin-c/d specific peptides with high mass response produced by trypsin digestion

Table S8: Common or specific marker peptides of four abrin isoforms

Table S9: The average MRM area (n=3) values of the peptides in matrix and matrix-free samples

Figure S2: Separation and purification of four isoforms of abrin by gel filtration chromatography.

Table S10: The relative content of different isoforms of abrin in the gel filtration fraction samples and abrin standard provided by OPCW

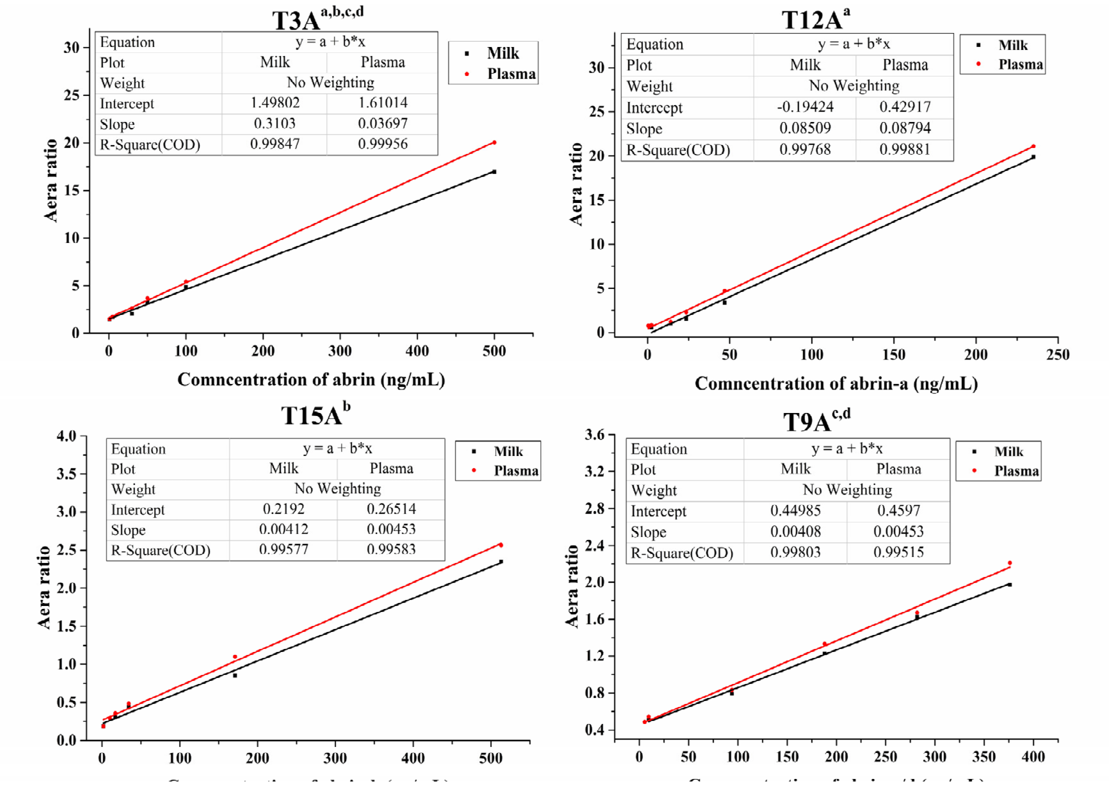

Figure S1. Standard curves of four type abrin peptide markers in milk (black) and plasma (red). Abrin (AS172.9) in increasing concentrations were spiked in milk and plasma. The samples were immunopurified using the prepared polyclonal coated IMBs. After the removal of unspecific bindings, ultrasound-assisted on-bead trypsin digestion was performed. Released peptides were quantified by LC-MS/MS with the developed MRM method and peak ratio with labeled peptides was illustrated. Furthermore, detection of the peptides of all abrin isoforms following IMBs enrichment with high sensitivity and linearity, indicating that the polyclonal antibody on can recognize all isoforms of abrin.

Table 1. Specific peptides of abrin-a generated by trypsin digestion.

T#&chain	Amino acid sequence	(M+H)⁺	(M+2H)²⁺	(M+3H)³⁺	(M+4H)⁴⁺
T1A[a]	QDRPIK	756.4363	378.7218	**-**	-
T2A[a]	FSTEGATSQSYK	1305.5957	**653.3015**	-	-
T3A[a,b,c,d]	QFIEALR	876.4938	**438.7505**	-	**-**
T6A[a]	GGLIHDIPVLPDPTTLQER	2071.1182	1036.0627	**691.0442**	518.5350
T9A[a]	AGTQSYFLR	1042.5316	**521.7694**	-	-
T11A[a]	WAHQSR	**784.3849**	392.6961	-	-
T12A[a]	QQIPLGLQALTHGISFFR	2026.1232	1013.5652	**676.0459**	507.2863
T13A[a]	SGGNDNEEK	949.3857	475.1965	-	-
T15A[a]	TLIVIIQMVAEAAR	1527.8927	**764.4500**	509.9691	-
T20A[a]	GVQESVQDTFPNQVTLTNIR	2246.1411	1123.5742	**749.3852**	562.2907
T21A-ss-T3B[a]	NEPVIVDSLSHPTVAVLALMLFVCNPPN-ss-ICSSR	3551.8058	1776.4066	1184.6068	**888.7069**
T4B[a,b]/T3B[c,d]	YEPTVR	764.3937	**382.7005**	-	-
T6B-ss-T8B[a,b]	DGMCVDVYDNGYHNGNR-ss-CK	2175.8640	1088.4357	725.9595	**544.7215**
T7B[a,b]	IIMWK	690.4007	**345.7040**	-	-
T10B[a,b]/T9B[c,d]	LEENQLWTLK	1273.6787	**637.3430**	**-**	-
T14B[a,b]	CLTTEGYAPGNYVMIYDCTSAVAEATYW EIWDNGTIINPK	4471.0243	982.9344	655.6254	-
T15B[a,b]/T14B[c]	SALVLSAESSSMGGTLTVQTNEYLMR	2745.3434	1373.1753	**915.7860**	687.0913
T19B[a,b]/T18[c]/T17B[d]	EQQWALYTDGSIR	1566.7547	**783.8810**	522.9231	-
T20B-ss-T22B[a]	SVQNTNNCLTSK-ss-QGSTILLMGCSNGWASQR	3214.5038	**1607.7556**	1072.1728	804.3814
T24B[a]/T22B[d]	NDGSIYSLYDDMVMDVK	1964.8616	982.9344	**655.6254**	-
T25B[a]/T23B[d]	GSDPSLK	**703.3621**	352.1847	**-**	-
T26B[a]/T24B[d]	QIILWPYTGKPNQIWLTLF	2331.2900	1166.1486	**777.7682**	583.5780

The LC-MS observed ions are indicated in bold; The abrin-a specific peptides were indicated in red

Table S2. Specific peptides of abrin-b generated by trypsin digestion.

T#&chain	Amino acid sequence	(M+H)$^{+}$	(M+2H)$^{2+}$	(M+3H)$^{3+}$	(M+4H)$^{4+}$
T1A[b,c,d]	QDQVIK	**730.4094**	365.7083	-	-
T2A[b,c,d]	FTTEGATSQSYK	1319.6114	**660.3093**	-	-
T3A[a,b,c,d]	QFIEALR	876.4938	**438.7505**	-	-
T5A[b]	LTGGLIHGIPVLPDPTTLQER	2227.2445	1114.1259	**743.0863**	557.5666
T10A[b]	DAPTSASR	**804.3846**	402.6959	-	-
T11A[b]	YLFTGTQQYSLR	1476.7482	**738.8777**	-	-
T12A[b]	FNGSYIDLER	1213.5848	607.2960	405.1998	-
T15A[b]	QQIPLGLQALR	1236.7423	**618.8748**	-	-
T16A[b]	HAISFLQSGTDDQEIAR	1887.9195	944.4634	**629.9780**	-
T17A[b,c,d]	TLIVIIQMASEAAR	1515.8563	**758.4318**	505.9569	-
T20A[b,c,d]	VGVSIR	630.3933	**315.7003**	-	-
T22A-ss-T3B[b]	SVNNQPVIVDSLTHQSVAVLALMLFVCNPP N-ss-ICSSR	3881.9710	1941.4892	1294.6619	**971.2482**
T4B[a,b]/T3B[c,d]	YEPTVR	764.3937	**382.7005**	-	-
T6B-ss-T8B[a,b]	DGMCVDVYDNGYHNGNR-ss-CK	2175.8640	1088.4357	725.9595	**544.7215**
T7B[a,b]	IIMWK	690.4007	**345.7040**	-	-
T10B[a,b]/T9B[c,d]	LEENQLWTLK	1273.6787	**637.3430**	-	-
T14B[a,b]	CLTTEGYAPGNYVMIYDCTSAVAEATYWE IWDNGTIINPK	4471.0243	982.9344	655.6254	-
T15B[a,b]/T14B[c]	SALVLSAESSSMGGTLTVQTNEYLMR	2745.3434	1373.1753	**915.7860**	687.0913
T19B[a,b]/T18[c]/T17B[d]	EQQWALYTDGSIR	1566.7547	**783.8810**	522.9231	-
T20B-ss-T22B[b]/ T19B-ss-T21B[c,d]	SVQNTNNCLTSK-ss- QGSPIVLMACSNGWASQR	3210.5100	1605.7587	1070.8415	**803.3830**
T24B[b]/T23B[c]	NDGSIYNLHDDMVMDVK	1965.8681	983.4377	**655.9609**	-
T26B[b]/T25B[c]	SDPSLK	646.3406	**323.6739**	-	-
T27B[b]/T26B[c]	EIILHPYHGKPNQIWLTLF	2319.2648	1160.1360	773.7598	580.5717

The LC-MS observed ions are indicated in bold; The abrin-b specific peptides were indicated in red

Table S3. Specific peptides of abrin-c generated by trypsin digestion.

T#&chain	Amino acid sequence	$(M+H)^+$	$(M+2H)^{2+}$	$(M+3H)^{3+}$	$(M+4H)^{4+}$
T1A[b,c,d]	QDQVIK	**730.4094**	365.7083	-	-
T2A[b,c,d]	FTTEGATSQSYK	1319.6114	**660.3093**	-	-
T3A[a,b,c,d]	QFIEALR	876.4938	**438.7505**	-	-
T5A[c,d]	LTGGLIHDIPVLPDPTTVEER	2272.2183	1136.6128	758.0776	568.8100
T7A[c,d]	YITVELSNSER	1310.6587	**655.8330**	-	-
T8A[c,d]	ESIEVGIDVTNAYVVAYR	1998.0178	999.5125	666.6774	500.2599
T9A[c,d]	AGSQSYFLR	1028.5160	**514.7616**	-	-
T10A[c,d]	DAPASASTYLFPGTQR	1681.8180	841.4126	561.2775	-
T12A[c,d]	FDGSYGDLER	1158.5062	**579.7567**	-	-
T13A[c,d]	WAHQTR	798.4006	399.7039	-	-
T14A[c,d]	EEISLGLQALTHAISFLR	1998.1018	999.5545	**666.7054**	500.2809
T15A[c,d]	SGASNDEEK	936.3905	468.6989	-	-
T17A[b,c,d]	TLIVIIQMASEAAR	1515.8563	**758.4318**	505.9569	-
T20A[b,c,d]	VGVSIR	630.3933	**315.7003**	-	-
T22A-ss-T2B[c,d]	QPVVVDSLSHPTVAVLALMLFVCNPPN-ss-ICSSR	3422.7632	1711.8853	1141.5926	856.4463
T1B[c,d]	IVEESK	704.3825	352.6949	-	-
T4B[a,b]/T3B[c,d]	YEPTVR	764.3937	**382.7005**	-	-
T5B-ss-T7B[c,d]	DGMCVDVYDDGYHNGNR-ss-CK	2176.8480	1088.9277	726.2875	**544.9675**
T10B[a,b]/T9B[c,d]	LEENQLWTLK	1273.6787	**637.3430**	-	-
T15B[a,b]/T14B[c]	SALVLSAESSSMGGTLTVQTNEYLMR	2745.3434	1373.1753	**915.7860**	687.0913
T19B[a,b]/T18[c]/T17B[d]	EQQWALYTDGSIR	1566.7547	**783.8810**	522.9231	-
T20B-ss-T22B[b]/ T19B-ss-T21B[c,d]	SVQNTNNCLTSK-ss-QGSPIVLMACSNGWASQR	3210.5100	1605.7587	1070.8415	**803.3830**
T24B[b]/T23B[c]	NDGSIYNLHDDMVMDVK	1965.8681	983.4377	**655.9609**	-
T26B[b]/T25B[c]	SDPSLK	646.3406	**323.6739**	-	-
T27B[b]/T26B[c]	EIILHPYHGKPNQIWLTLF	2319.2648	1160.1360	**773.7598**	580.5717

The LC-MS observed ions are indicated in bold; The abrin-c specific peptides were indicated in red

Table S4. Specific peptides of abrin-d generated by trypsin digestion.

T#&chain	Amino acid sequence	$(M+H)^+$	$(M+2H)^{2+}$	$(M+3H)^{3+}$	$(M+4H)^{4+}$
T1A[b,c,d]	QDQVIK	730.4094	365.7083	-	-
T2A[b,c,d]	FTTEGATSQSYK	1319.6114	**660.3093**	-	-
T3A[a,b,c,d]	QFIEALR	876.4938	438.7505	-	-
T5A[c,d]	LTGGLIHDIPVLPDPTTVEER	2272.2183	1136.6128	758.0776	**568.8100**
T7A[c,d]	YITVELSNSER	1310.6587	**655.8330**	-	-
T8A[c,d]	ESIEVGIDVTNAYVVAYR	1998.0178	999.5125	666.6774	500.2599
T9A[c,d]	AGSQSYFLR	1028.5160	**514.7616**	-	-
T10A[c,d]	DAPASASTYLFPGTQR	1681.8180	841.4126	561.2775	-
T12A[c,d]	FDGSYGDLER	1158.5062	**579.7567**	-	-
T13A[c,d]	WAHQTR	798.4006	**399.7039**	-	-
T14A[c,d]	EEISLGLQALTHAISFLR	1998.1018	999.5545	**666.7054**	500.2809
T15A[c,d]	SGASNDEEK	936.3905	468.6989	-	-
T17A[b,c,d]	TLIVIIQMASEAAR	1515.8563	758.4318	**505.9569**	-
T20A[b,c,d]	VGVSIR	630.3933	**315.7003**	-	-
T22A-ss-T2B[c,d]	QPVVVDSLSHPTVAVLALMLFVCNPPN-ss-ICSSR	3422.7632	1711.8853	**1141.5926**	856.4463
T1B[c,d]	IVEESK	704.3825	352.6949	-	-
T4B[a,b]/T3B[c,d]	YEPTVR	764.3937	**382.7005**	-	-
T5B-ss-T7B[c,d]	DGMCVDVYDDGYHNGNR-ss-CK	2176.8480	1088.9277	**726.2875**	544.9675
T10B[a,b]/T9B[c,d]	LEENQLWTLK	1273.6787	**637.3430**	-	-
T19B[a,b]/T18[c]/T17B[d]	EQQWALYTDGSIR	1566.7547	**783.8810**	522.9231	-
T20B-ss-T22B[b]/T19B-ss-T21B[c,d]	SVQNTNNCLTSK-ss-QGSPIVLMACSNGWASQR	3210.5100	**1605.7587**	1070.8415	803.3830
T24B[a]/T22B[d]	NDGSIYSLYDDMVMDVK	1964.8616	**982.9344**	655.6254	-
T25B[a]/T23B[d]	GSDPSLK	703.3621	**352.1847**	-	-
T26B[a]/T24B[d]	QIILWPYTGKPNQIWLTLF	2331.2900	**1166.1486**	777.7682	583.5780

The LC-MS observed ions are indicated in bold; The abrin-d specific peptides were indicated in red

Table S5. Abrin-a specific peptides with high mass response produced by trypsin digestion.

T#&chain	**Amino acid sequence**
T15A[a]	TLIVIIQMVAEAAR
T3A[a,b,c,d]	QFIEALR
T4B[a,b]/T3B[c,d]	YEPTVR
T12A[a]	QQIPLGLQALTHGISFFR
T9A[a]	AGTQSYFLR
T6A[a]	GGLIHDIPVLPDPTTLQER
T19B[a,b]/T18[c]/T17B[d]	EQQWALYTDGSIR
T2A[a]	FSTEGATSQSYK
T11A[a]	WAHQSR
T7B[a,b]	IIMWK
T20A[a]	GVQESVQDTFPNQVTLTNIR
T10B[a,b]/T9B[c,d]	LEENQLWTLK

Arrange peptides top to bottom based on the mass spectrum response; The abrin-a specific peptides were indicated in red

Table S6. Abrin-b specific peptides with high mass response produced by trypsin digestion.

**T#&chain**	**Amino acid sequence**
T17A[b,c,d]	TLIVIIQMASEAAR
T3A[a,b,c,d]	QFIEALR
T20A[b,c,d]	VGVSIR
T4B[a,b]/T3B[c,d]	YEPTVR
T19B[a,b]/T18[c]/T17B[d]	EQQWALYTDGSIR
T15A[b]	QQIPLGLQALR
T5A[b]	LTGGLIHGIPVLPDPTTLQER
T7B[a,b]	IIMWK
T1A[b,c,d]	QDQVIK
T24B[b]/T23B[c]	NDGSIYNLHDDMVMDVK
T16A[b]	HAISFLQSGTDDQEIAR
T2A[b,c,d]	FTTEGATSQSYK
T10B[a,b]/T9B[c,d]	LEENQLWTLK

Arrange peptides top to bottom based on the mass spectrum response; The abrin-b specific peptides were indicated in red

Table S7. Abrin-c/d specific peptides with high mass response produced by trypsin digestion.

**T#&chain**	**Amino acid sequence**
T17A[b,c,d]	TLIVIIQMASEAAR
T3A[a,b,c,d]	QFIEALR
T20A[b,c,d]	VGVSIR
T4B[a,]b/T3B[c,d]	YEPTVR
T19B[a,b]/T18[c]/T17B[d]	EQQWALYTDGSIR
T1A[b,c,d]	QDQVIK
T24B[b]/T23B[c]	NDGSIYNLHDDMVMDVK
T2A[b,c,d]	FTTEGATSQSYK
T10B[a,b]/T9B[c,d]	LEENQLWTLK
T24B[a]/T22B[d]	NDGSIYSLYDDMVMDVK
T9A[c,d]	AGSQSYFLR
T12A[c,d]	FDGSYGDLER
T14A[c,d]	EEISLGLQALTHAISFLR
T7A[c,d]	YITVELSNSER

Arrange peptides top to bottom based on the mass spectrum response; The abrin-c/d specific peptides were indicated in red

Table S8. Common or specific marker peptides of four abrin isoforms.

T#&chain	Amino acid sequence	(M+H)$^{+}$	(M+2H)$^{2+}$	(M+3H)$^{3+}$	(M+4H)$^{4+}$
T3A[a,b,c,d]	QFIEALR	876.4938	**438.7505**	-	-
T4B[a,b]/T3B[c,d]	YEPTVR	764.3937	**382.7005**	-	-
T10B[a,b]/T9B[c,d]	LEENQLWTLK	1273.6787	**637.3430**	-	-
T19B[a,b]/T18[c]/T17B[d]	EQQWALYTDGSIR	1566.7547	**783.8810**	522.9231	-
T2A[a]	FSTEGATSQSYK	1305.5957	**653.3015**	-	-
T6A[a]	GGLIHDIPVLPDPTTLQER	2071.1182	1036.0627	691.0442	**518.5350**
T9A[a]	AGTQSYFLR	1042.5316	**521.7694**	-	-
T12A[a]	QQIPLGLQALTHGISFFR	2026.1232	1013.5652	**676.0459**	507.2863
T5A[b]	LTGGLIHGIPVLPDPTTLQER	2227.2445	1114.1259	**743.0863**	557.5666
T15A[b]	QQIPLGLQALR	1236.7423	**618.8748**	-	-
T16A[b]	HAISFLQSGTDDQEIAR	1887.9195	944.4634	**629.9780**	-
T7A[c,d]	YITVELSNSER	1310.6587	**655.8330**	-	-
T9A[c,d]	AGSQSYFLR	1028.5160	**514.7616**	-	-
T12A[c,d]	FDGSYGDLER	1158.5062	**579.7567**	-	-
T14A[c,d]	EEISLGLQALTHAISFLR	1998.1018	999.5545	**666.7054**	500.2809

The LC-MS observed ions are indicated in bold

Table S9. The average MRM area (n=3) values of the peptides in matrix and matrix-free samples.

Sample ID	Matrices	Spiking Concertation of peptides (ng/mL)	Average value of MRM area of T3A[a,b,c,d]	Average value of MRM area of T12A[a]	Average value of MRM area of T15A[b]	Average value of MRM area of T9A[c,d]
QC-L	H_2O	2.00	1838	598	136	1031
QC-M	H_2O	20.0	19832	6630	1499	11209
QC-H	H_2O	100	52197	12103	3038	20547
QC-L	Milk	2.00	2367	662	169	1391
QC-M	Milk	20.0	21061	575	131	943
QC-H	Milk	100	53919	11413	2944	21102
QC-L	Plasma	2.00	2068	676	171	1143
QC-M	Plasma	20.0	22351	6451	1626	10996
QC-H	Plasma	100	47969	11232	2295	19828

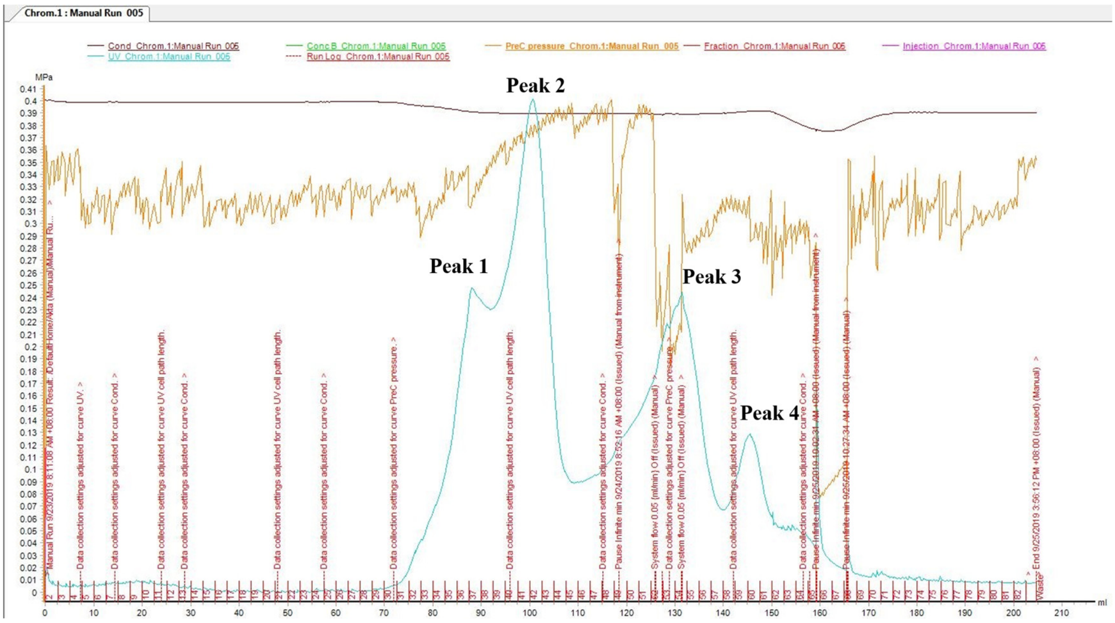

Figure S2. Separation and purification of four isoforms of abrin by gel filtration chromatography; The abscissa is the elution volume, the ordinate is the absorbance, the blue is the absorbance curve of abrin, and the brown is the ion concentration.

Table S10. The relative content of different isoforms of abrin in the gel filtration fraction samples and abrin standard.

Sample ID	Gel filtration chromatography peak	Fraction	Abrin-a content	Abrin-b content	Abrin-c/d content
1	Peak 1	32	23.1%	44.9%	32.0%
2		34	33.8%	40.6%	25.6%
3		35	50.4%	31.0%	18.6%
4		37	54.2%	28.6%	17.2%
5	Peak 2	40	2.3%	30.8%	47.8%
6		42	7.3%	50.2%	42.5%
7		43	11.5%	55.8%	32.8%
8	Peak 3	53	100%	-	-
9		54	100%	-	-
10		55	100%	-	-
11		57	100%	-	-
12	Peak 4	60	81.3%	18.7%	-
13		61	79.5%	20.5%	-
14		65	77.0%	23.0%	-
15	standard	AS172.9*	47%	34.2%	18.8%

*: Abrin standards provided by the OPCW biotoxin analysis exercise
